# Bone-inspired (GNEC/HAPAAm) hydrogel with fatigue-resistance for use in underwater robots and highly piezoresistive sensors

**DOI:** 10.1038/s41378-023-00571-7

**Published:** 2023-07-26

**Authors:** Chaoyang Lyu, Bo Wen, Yangzhen Bai, Daning Luo, Xin Wang, Qingfeng Zhang, Chenyang Xing, Tiantian Kong, Dongfeng Diao, Xi Zhang

**Affiliations:** 1 Institute of Nanosurface Science and Engineering, Guangdong Provincial Key Laboratory of Micro/Nano Optomechatronics Engineering, Shenzhen University, 518060 Shenzhen, China; 2 Research Center of Medical Plasma Technology, Shenzhen University, 518060 Shenzhen, China; 3 Key Laboratory of Optoelectronic Devices and Systems of Ministry of Education, College of Physics and Optoelectronic Engineering, Shenzhen University, 518060 Shenzhen, China; 4 Department of Biomedical Engineering, School of Medicine, Shenzhen University, 518000 Shenzhen, China

**Keywords:** Nanoscale devices, Nanoscale materials

## Abstract

A novel bone-inspired fatigue-resistant hydrogel with excellent mechanical and piezoresistive properties was developed, and it exhibited great potential as a load and strain sensor for underwater robotics and daily monitoring. The hydrogel was created by using the high edge density and aspect ratio of graphene nanosheet-embedded carbon (GNEC) nanomaterials to form a three-dimensional conductive network and prevent the expansion of microcracks in the hydrogel system. Multiscale progressive enhancement of the organic hydrogels (micrometer scale) was realized with inorganic graphene nanosheets (nanometer scale). The graphene nanocrystals inside the GNEC film exhibited good electron transport properties, and the increased distances between the graphene nanocrystals inside the GNEC film caused by external forces increased the resistance, so the hydrogel was highly sensitive and suitable for connection to a loop for sensing applications. The hydrogels obtained in this work exhibited excellent mechanical properties, such as tensile properties (strain up to 1685%) and strengths (stresses up to 171 kPa), that make them suitable for use as elastic retraction devices in robotics and provide high sensitivities (150 ms) for daily human monitoring.

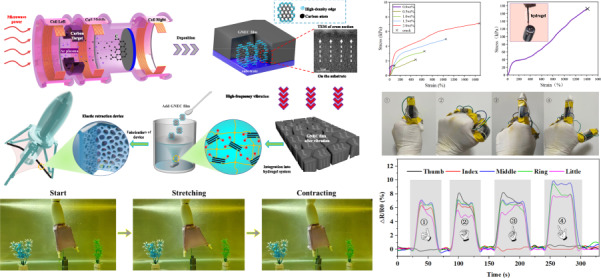

## Introduction

In many popular engineering applications, such as underwater robotic elastic retraction devices and flexible wearable sensing parts, flexible materials are needed. Hydrogels have three-dimensional reticular cross-linked structures and are ideal choices for this purpose. In recent years, hydrogels have undergone rapid development and have been applied in various fields, such as flexible wearable sensors^1–7^, cartilage tissues^8,9^, and agricultural fertilization^10^. However, their low tensile strengths and poor elongations at break limit their use in engineering^11^.

As reported, hydrogels typically have low tensile strengths due to the limitations of the polymer’s mechanical properties, which makes them unsuitable for strength-bearing uses. However, researchers have proposed several strategies to improve the strain capacities of hydrogels. For example, hydrogels composed of interpenetrating sodium carboxymethylcellulose microsheets and a polyacrylamide network showed fracture tensile strengths of up to 23 kPa and strains of up to 1400%^12^. Gelatin-based dual-network hydrogels with multiple dynamic cross-linking exhibited 25 kPa fracture tensile strengths and 1200% strains^13^. Hydrogels composed of lignin-based carbon, polyvinyl alcohol, carboxymethyl chitosan and cellulose nanofibers reached a tensile strength of 133 kPa but only reached a strain of 320%^14^. Currently, the addition of carbon or carbon-based materials to enhance the mechanical properties of hydrogels is also a popular strategy. Hydrogels containing CNTs^15–20^ have tensile strengths ranging from 6 to 160 kPa and tensile strains ranging from 200 to 600%. Among them, the hydrogel with the highest tensile strength, 160 kPa, had a tensile strain of less than 300%^18^. The hydrogel with the highest tensile strain, 600%, exhibited a tensile strength of less than 30 kPa^19^. Hydrogels containing graphene^21–26^, most of which have tensile strengths in the range 30–100 kPa and tensile strains in the range 250–1300%. Among them, the hydrogel with the highest tensile strength, 100 kPa, had a tensile strain of less than 250%^21^. The highest tensile strain of a hydrogel was 1300%, but its tensile strength was less than 70 kPa^24^. Hydrogels containing MXenes^27–31^ have tensile strengths of 12 kPa to 100 kPa and tensile strains of 700–900%. Among them, the highest tensile strength was 100 kPa, but the tensile strain of that hydrogel was less than 800%^29^. The highest tensile strain for a hydrogel was 900%, but its tensile strength was less than 40 kPa^30^. When attempting to improve the tensile strengths of hydrogels, their strain capacities are often sacrificed. Therefore, it is necessary to find a method to increase both the tensile strengths and the tensile strains of hydrogels.

Ordinary hydrogels are typically electrical insulators. However, different methods have been used to improve the electrical conductivities of hydrogels, such as in ionic liquid segmental hydrogels consisting of acrylic acid^32,33^ or micrometer-sized silver flakes^34^. The electrical properties of hydrogels are commonly enhanced by adding carbon materials or carbon-based materials. Hydrogels^1,29,35–39^ containing CNTs, graphene or MXenes have exhibited good electrical properties, and most of them have a response range of 200 ms to 600 ms. Balancing the mechanical and electrical properties of hydrogels is challenging. Recently, graphene nanosheet-embedded carbon (GNEC) films were proposed to have vertically grown graphene nanosheets^40^ and high-density edges and the unique ability to trap electrons. This three-dimensional conductive network with hybrid properties between those of organic and inorganic materials may be responsible for the enhanced electrical and mechanical properties of the GNEC-filled hydrogels. To ensure good mechanical properties while improving electrical conductivity, the addition of inorganic carbon nanomaterials is a good choice.

By taking inspiration from nature, we designed a stretchable, fatigue-resistant, conductive, and highly sensitive composite hydrogel by integrating a graphene nanosheet-embedded carbon (GNEC) film into a hydrophobic associative polyacrylamide hydrogel (HAPAAm). During the polymerization process in water, a porous bone-like structure was formed, which greatly improved the conductive network and enhanced the tensile strength of the hydrogel. Sodium dodecyl sulfate (SDS) was used to ensure uniform dispersion of the GNEC film in the hydrogel network and provide sites for cross-linking between the poly(lauryl methacrylate) chain segments. The introduction of PLMA chain segments further improved the interfacial interactions between the GNEC film and the hydrogel matrix. The GNEC film enhanced the mechanical properties of the hydrogel while improving its electrical conductivity. This composite hydrogel containing the GNEC film is expected to have excellent mechanical properties and good electrical conductivity and can be used as an elastic retraction device and sensor. Our results showed that the prepared GNEC/HAPAAm hydrogel exhibited excellent tensile and fatigue resistance as well as electrical conductivity and sensitivity.

## Experimental section

### Preparation of the GNEC film

The graphene nanosheet-embedded carbon (GNEC) film we used was prepared with an electron cyclotron resonance (ECR) sputtering system. In detail, the SiO_2_ substrate was degreased with acetone, cleaned with anhydrous ethanol, and placed in the vacuum chamber of the electron cyclotron resonance (ECR) sputtering system. The vacuum cavity was maintained at a pressure of 3 × 10^−4^ Pa, and argon gas was introduced to maintain the atmospheric pressure at 0.04 Pa. The substrate surface was cleaned with an Ar plasma for three minutes prior to depositing the GNEC film. After cleaning, the GNEC film was deposited with a substrate bias voltage of 80 V. Microwave power was delivered into the vacuum chamber to generate a plasma, and a magnetic field was applied to enhance the plasma density. In a high vacuum argon ion (Ar^+^) plasma atmosphere, the carbon atoms of the carbon target were bombarded by the plasma ions. Meanwhile, the positive deposition bias (V_dep_ = 80 V) attracted electrons to the substrate. After acceleration, the active electrons provided the formation energy for sp²bonds in the carbon nanostructure. High-density graphene nanocrystals grew vertically on the SiO_2_ substrate. After deposition, the substrate was removed from the vacuum chamber. The GNEC film was mechanically peeled off from the SiO_2_ substrate, and shattered into a powder (with particle sizes of 10 μm) by a high-frequency vibration device.

### Preparation of the GNEC/HAPAAm hydrogel

First, 129.8 mg of the GNEC film powder (wt% = 2.0%), 150 mg of SDS and 150 μl of LMA were added to 30 ml of deionized water and stirred rapidly at room temperature. After 1 h, the mixed solution was irradiated with ultrasonic waves for 30 min. Then, 6.36 g of AAm was added to the solution and stirred for another 20 min. After stirring, 20.4 mg of APS and 60 μl of TEMED were added to the solution and stirred for another 10 min. Finally, the resulting solution was transferred to a pre-prepared mold. The polymerization reaction occurred within 10 min and provided the GNEC/HAPAAm hydrogel. Among the reagents used, the GNEC film powder content can be adjusted according to the actual demand, and the GNEC film powder contents (wt%) used in this work were 0, 0.5%, 1.0%, 1.5%, and 2.0%. The GNEC film powder content used in preparing the hydrogel was calculated as ${\rm{wt}} \% =\frac{{{\rm{m}}}_{{\rm{C}}}}{{{\rm{m}}}_{{\rm{C}}}+{{\rm{m}}}_{{\rm{A}}}}$, where ${{\rm{m}}}_{{\rm{C}}}$ is the mass of the carbon film and ${{\rm{m}}}_{{\rm{A}}}$ is the mass of AAm (acrylamide).

### Materials

The GNEC film was from the Institute of Nanosurface Science and Engineering (Shenzhen, China). Acrylamide (AAm, 99%), ammonium persulfate (APS, 98%), and tetramethylethylenediamine (TEMED, 99%) were purchased from Aladdin (Shanghai, China). Sodium dodecyl sulfate (SDS, 99%) and lauryl methacrylate (LMA, 96%) were purchased from Rohn (Shanghai, China). Deionized water was prepared prior to the experiments.

### SEM

The internal microstructures of the GNEC/HAPAAm hydrogels were observed by scanning electron microscopy (FEI Scios). Before observation, the hydrogel samples were broken in liquid nitrogen, freeze-dried, and finally observed by SEM.

### TEM

The nanostructures of the GNEC films were observed by transmission electron microscopy (FEI Titan Cubed Themis G2 300).

### Raman

The Raman spectra were obtained with a Horiba HR800 Evolution system with an excitation laser wavelength of 532 nm.

### FIB

A cross-section sample of the 80 V GNEC film on the substrate was prepared by focused ion beam (FIB) etching. The protective layer was a layer of platinum with a thickness of 2 μm, which was deposited on the surface of the sample with an auxiliary gas injection system associated with the FIB-SEM system prior to the FIB etching process. The lateral thickness of the cross-section sample was etched to less than 100 nm. We prepared a cross-section sample with a thickness of ~30 nm, which was more conducive to our observation of the vertical growth structure of the GNs by TEM. The transmission electron microscopy (TEM) image of the cross-section sample indicated that the GNs grew vertically on the silicon substrate with vertical thicknesses of ~70 nm.

### Mechanical properties

The mechanical properties, such as the stress-strain curves of the hydrogels with different GNEC film contents, were determined with a high-precision digital tensimeter (Shen Ce SC).

### Electrical properties

The electrical properties, such as the I–V curves of the hydrogels, were measured with a digital sourcemeter (Keithley 2400).

## Results and discussion

**Fig. 1:**
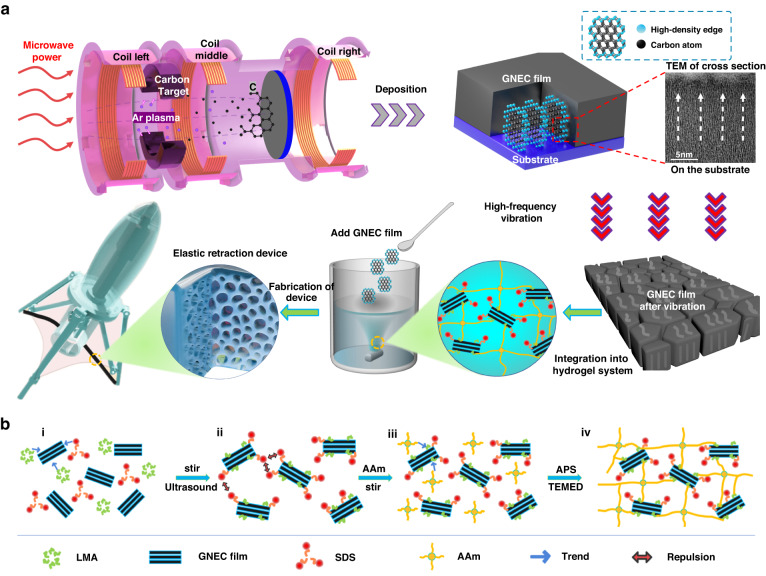
Preparation of a bone-like GNEC/HAPAAm hydrogel. **a** Preparation of GNEC film with the ECR sputtering system; shattering of the GNEC film into a powder (with particle sizes of 10 μm) with high-frequency vibration; mixing of the GNEC powder into the hydrogel solution to form the GNEC/HAPAAm hydrogel; fabrication of elastic retraction device for an underwater robot. **b** Schematic illustration of the preparation of the GNEC/HAPAAm hydrogel: (i) absorption of LMA and SDS on the GNEC film; (ii) dispersion of the GNEC film by repulsion with the assistance of SDS; (iii) addition of AAM monomers; and (iv) cross-linking of AAM and GNEC with the assistance of APS and TEMED

Hydrogels are used as elastic retraction devices in some mechanical devices, which require good mechanical properties. To improve the mechanical properties of the hydrophobic associative polyacrylamide (HAPAAm) hydrogel, we integrated graphene nanosheet-embedded carbon (GNEC) into the hydrogel system. As shown in Fig. 1a, preparation of the bone-like GNEC/HAPAAm hydrogel was divided into four stages. First, we prepared a GNEC film with an ECR sputtering system and a substrate bias of 80 V^41,42^. With the assistance of low-energy electrons, graphene nanosheets are grown vertically on the SiO_2_ substrate^43^, as shown in the TEM image of the cross-section sample (made with a focused ion beam (FIB)). Second, we mechanically peeled the GENC film off the substrate and shattered the GNEC film into a powder (with particles sizes of 10 μm) with a high-frequency vibration device. Third, we mixed the GNEC powder into the hydrogel solution to prepare the GNEC/HAPAAm hydrogel. This step was further divided into four substages, as shown in Fig. 1b. In this process, SDS was used as a dispersant to disperse the GNEC film, while LMA was added to form a functional cross-linker. (i) SDS was absorbed on the surface of the GNEC film via hydrophobic interactions and exposed the hydrophilic sulfate fraction. In addition, some of the hydrophobic monomers, LMA, were absorbed on the GNEC film surface and partially dissolved inside the SDS, all of which acted as cross-linking agents through hydrophobic association^35^. (ii) SDS facilitated uniform dispersion of the GNEC film throughout the hydrogel system. (iii) AAm monomers were added to the solution. (iv) After adding the AAm, APS and TEMED initiators were used to copolymerize AAm with the other substances to form hydrogel bodies. This porous network structure filled with inorganic materials had strain characteristics similar to those of bones and improved the tensile strength of the hydrogel. Finally, we fabricated an elastic retraction device on an underwater robot.

**Fig. 2:**
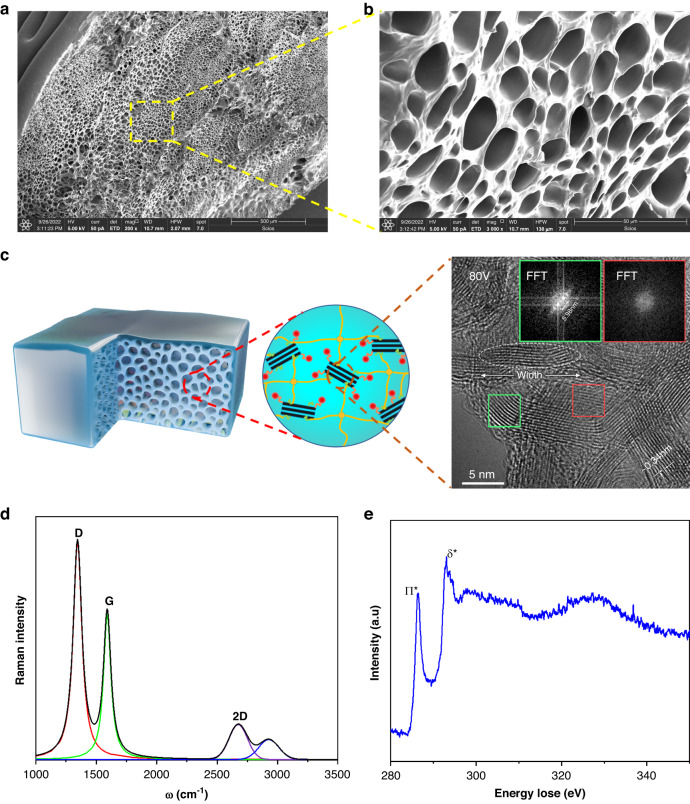
Microstructural characterizations of the GNEC film and GNEC/HAPAAm hydrogel. **a**, **b** SEM images of the GNEC/HAPAAm hydrogel (scales 500 μm and 50 μm). **c** TEM image of the GNEC film (scale 5 nm). **d** Raman spectrum of the GNEC film. **e** EELS of the GNEC film

Scanning electron microscopy images (SEM, Fig. 2a, b) revealed the microstructure of the GNEC/HAPAAm hydrogel, and the internal bone-like pore structure of the hydrogel was observed. The transmission electron microscopy images (TEM, Fig. 2c) showed the nanoscale structure of the GNEC film, which was fabricated with an electron cyclotron resonance sputtering system after depositing the GNEC film on a hard substrate, peeling, and grinding. During the electron cyclotron resonance sputtering process, the low-energy electrons exchanged energy with the valence electrons of the carbon atoms through inelastic scattering, resulting in a change in the C hybridization from sp^3^ to sp^2^ ^44^. This increased electron energy loss was essential in breaking the C–C bonds and generating sp^2^ hybridization. Many graphene nanocrystals embedded in the amorphous carbon grew perpendicular to the substrate with the assistance of low-energy electrons. By varying the electron irradiation energy during electron cyclotron resonance sputtering, we adjusted the nanostructure of the GNEC film. The embedded graphene nanocrystals provided a high-density edge to the GNEC film, and the significant improvement seen in the mechanical properties of the GNEC/HAPAAm hydrogel was also attributed to the high-density graphene edge of the GNEC film. As shown in Fig. 2d, Raman spectra of the GNEC film were used to characterize the structural defects and edges, the number of graphene layers, and the stacking pattern of the graphene layers. The ratio of the D-peak to G-peak reflected the edge structure of the GNEC film, and the 2D-peak reflected the crystallinity of the GNEC film and indicated the presence of graphene layers with amorphous structures, low crystallinities, and high-density edge structures. To confirm the presence of sp^2^ hybridization, we determined the electron energy loss spectrum of the GNEC film, as shown in Fig. 2e. In the high-energy part of the EELS spectrum, the π* peak appeared near 286 eV, which indicated the presence of sp^2^ bonds. In addition, the δ* peak appeared near 295 eV, which indicated the presence of a crystalline structure^45^.

**Fig. 3:**
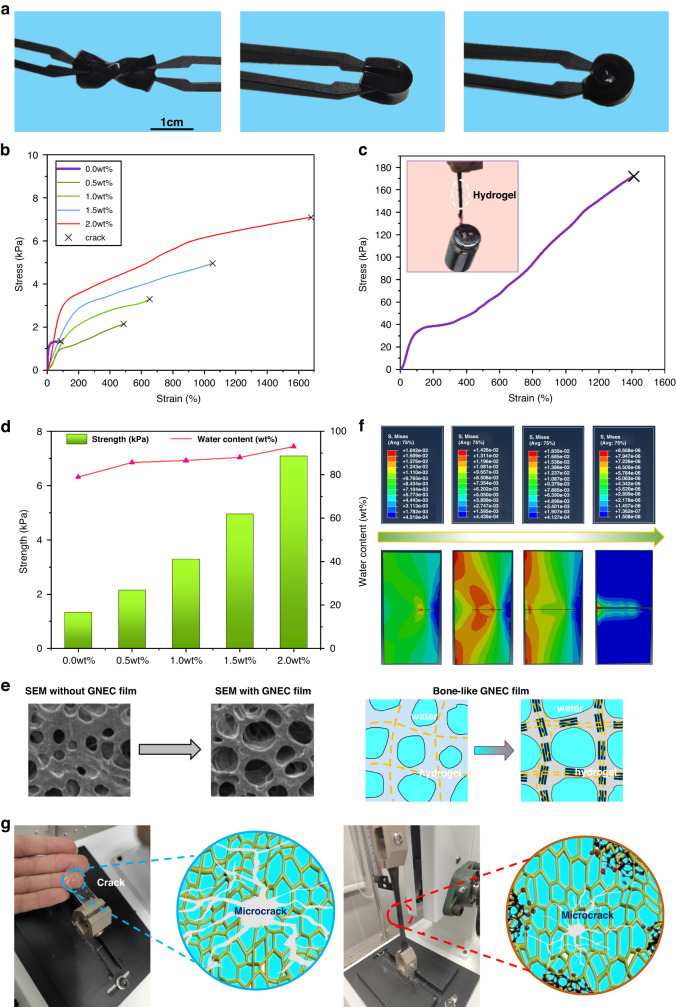
Mechanical properties of the GNEC/HAPAAm hydrogels. **a** Images showing hydrogel twisting, folding and curling. **b** Stress-strain curves of the hydrogels with different GNEC film contents. **c** Stress-strain curve for the GNEC/HAPAAm hydrogel (wt% = 2.0%) with a 40% water content. **d** Summary of the tensile strengths and water contents of hydrogels with different GNEC film contents. **e** Water content enhancement due to bone-like GNEC film. **f** A precracking simulation based on extended finite element analysis (performed by Abaqus). **g** Elongation at break and the microcrack propagation mechanism of the GNEC/HAPAAm hydrogel (microcrack propagation was prevented by the GNEC film)

Figure 3a shows the state of the GNEC/HAPAAm hydrogel when it was twisted, folded, and curled, and it exhibited good elasticity and flexibility. The macroscopic mechanical properties of the GNEC/HAPAAm hydrogels were measured with a tensile table. As shown in Fig. 3b, the tensile experiments were conducted on hydrogels with different GNEC film contents, where 0 wt% indicates the HAPAAm hydrogel. The analysis of the resulting stress-strain curves showed that the mechanical properties of this hydrogel were significantly enhanced with increasing GNEC film content, and the stress limit rose from 1.4 to 6.5 kPa and the strain degree increased from 95 to 1685%. This was attributed to the high density of the GNEC film edge. As shown in Fig. 3c, the stress limit (for a GNEC film content = 2.0 wt%) increased ~30 times with decreases in the water content (compared with Fig. 3b) and reached 171 kPa at 40% water content, when the hydrogel could bear a 500 g weight. Figure 3d shows that the water content of this hydrogel increased with increasing GNEC film content. The mechanism for the increases in water content is illustrated in Fig. 3e, which shows that the three-dimensional pore structure within the hydrogel system became larger due to the presence of the GNEC film, thus enabling it to retain more water. The water content of the hydrogel was calculated from the difference between the masses of the hydrogel in the equilibrium swollen state and the fully dried state with the formula ${\rm{water\; content}}=\frac{{{\rm{m}}}_{{\rm{w}}}-{{\rm{m}}}_{{\rm{d}}}}{{{\rm{m}}}_{{\rm{w}}}}$, where ${{\rm{m}}}_{{\rm{w}}}$ is the mass of the hydrogel in the equilibrium swollen state and ${{\rm{m}}}_{{\rm{d}}}$ is the mass of the hydrogel in the fully dried state. As shown in Fig. 3f, precracking of the GNEC/HAPAAm hydrogel was modeled with extended finite element simulation based on the parameters for a GNEC film content of 2.0 wt%. The images revealed the real-time force distribution of the hydrogel with precutting during the stretching process, and it was observed that when the cut was made under an applied force, there was a force that prevented bracket growth, which came from the GNEC film. It was also verified that the GNEC/HAPAAm hydrogel had an energy dissipation mechanism. As shown in Fig. 3g, when the hydrogel system was subjected to an external tensile force and microcracks were generated, the GNEC film effectively prevented crack extension, while the hydrogel system without the GNEC film was easily stretched to fracture.

**Fig. 4:**
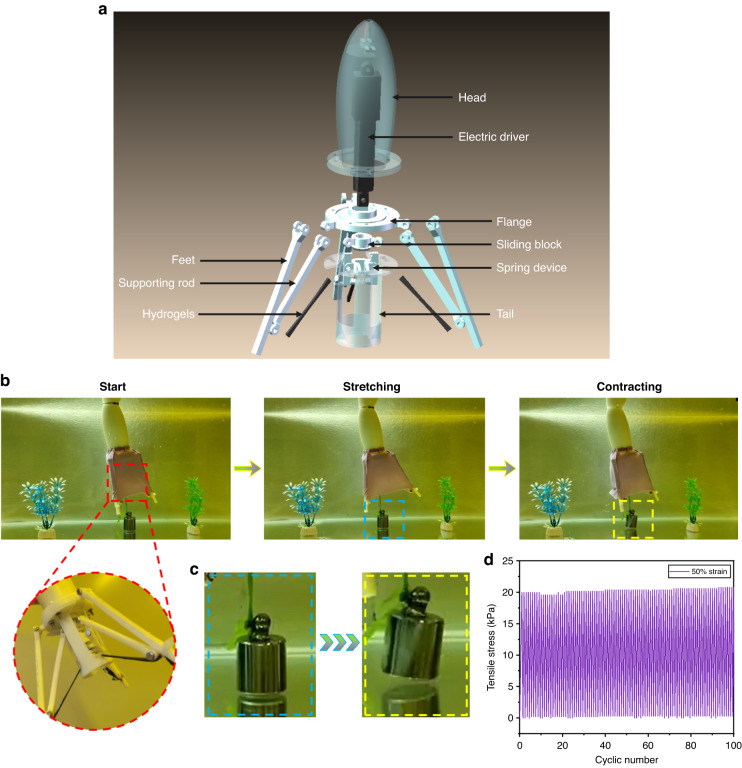
Demonstration of applications with the underwater robot. **a** Exploded view of the robot structure. **b** Swimming action of the underwater robot. **c** Displacement of the weight carried by the underwater robot. **d** Cyclic tensile tests of the GNEC/HAPAAm hydrogel

It was observed that the strength of the GNEC/HAPAAm hydrogel was ~30 times stronger with a water content of ~40% than it was when freshly fabricated (with a water content of ~85%), so this hydrogel has potential as an elastic retraction device for underwater robots and other applications. As shown in Fig. 4a, we designed and fabricated an underwater robot that used an electric actuator to expand the feet. The GNEC/HAPAAm hydrogel retracted elastically and pushed the surrounding water to generate thrust, which allowed the robot to swim underwater and carry a 100 g weight, as shown in Fig. 4b, c. Furthermore, the GNEC/HAPAAm hydrogel was subjected to 100 tensile tests with a 50% strain, and the tensile stress of this hydrogel was almost constant during continuous stretching cycles, indicating that this hydrogel has good fatigue resistance (Fig. 4d).

**Fig. 5:**
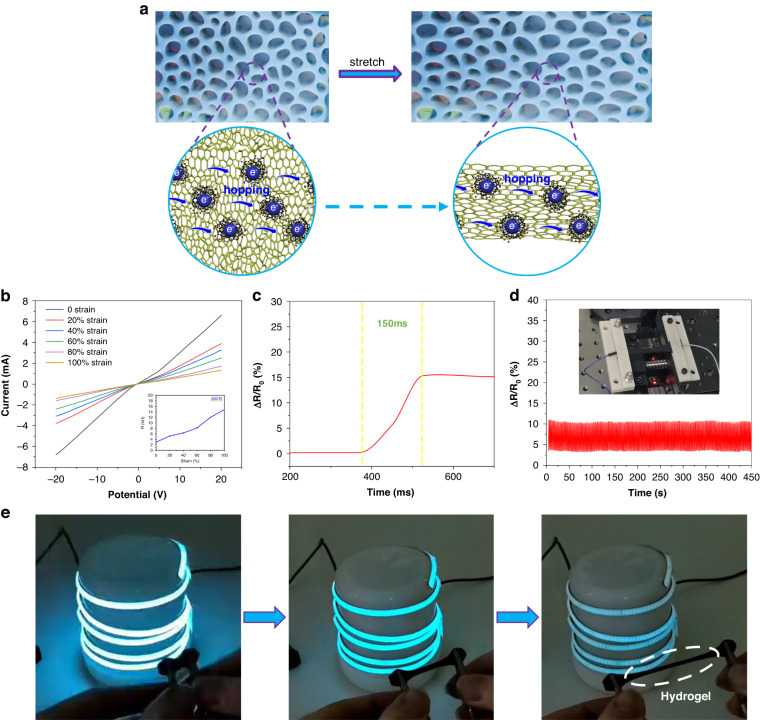
Electrical properties of the GNEC/HAPAAm hydrogels. **a** Schematic for electron hopping in the conductive network of the GNEC/HAPAAm hydrogel. **b** I–V curves for the GNEC/HAPAAm hydrogel at different strain states. **c** Response rate of the GNEC/HAPAAm hydrogel. **d** Stability test of the GNEC/HAPAAm hydrogel at 10% strain. **e** Stretching of the GNEC/HAPAAm hydrogel leading to a change in light brightness

As shown in Fig. 5a, the overall electron mobility of the hydrogel system decreased as the hydrogel was stretched with an external force. Figure 5b shows that the hydrogel (GNEC film content of 2.0 wt%) was stretched to different strain states, and its I–V curves showed significant changes confirming that the hydrogel had good electrical properties. In addition, Fig. 5c reveals that the GNEC/HAPAAm hydrogel had a good response speed, up to 150 ms, which would meet the requirements for detecting daily human motions. Furthermore, Fig. 5d demonstrates that the GNEC/HAPAAm hydrogel maintained a stable rate of resistance change under a 10% tensile strain and for ~500 stretching cycles, indicating that the hydrogel was highly stable. The formula used to calculate the rate of the resistance change was $\triangle {{\rm{R}}/{\rm{R}}}_{0}=\frac{{\rm{R}}-{{\rm{R}}}_{0}}{{{\rm{R}}}_{0}}\times 100 \%$, where ${{\rm{R}}}_{0}$ is the initial resistance of the hydrogel sample when it is not stretched, and ${\rm{R}}$ is the real-time resistance of the hydrogel sample during the stretching process. Finally, Fig. 5e shows that the GNEC/HAPAAm hydrogel was connected into a loop with an LED light, and the hydrogel was stretched continuously. Significant changes were observed in the brightness of the blue LED light, which demonstrated the sensitivity and response time of the hydrogel.

**Fig. 6:**
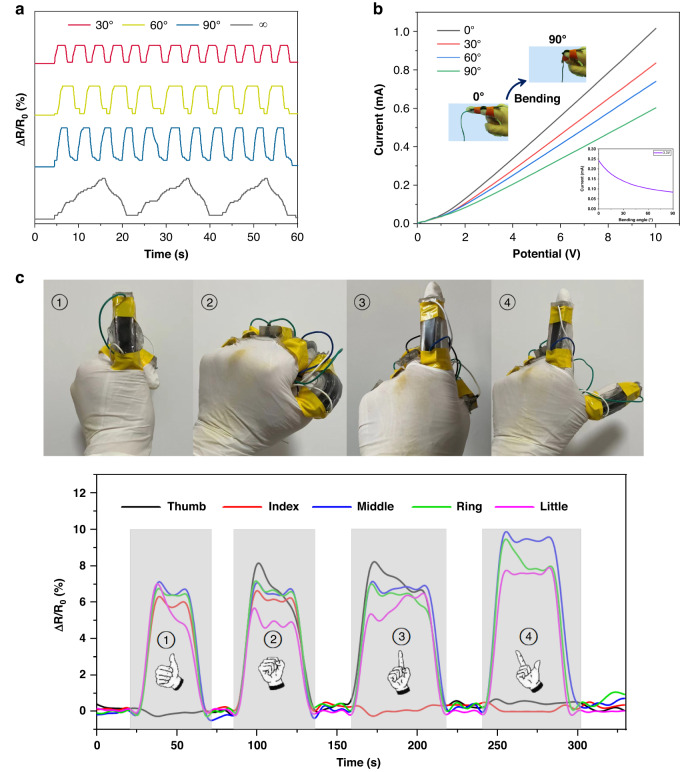
Sensing applications for hand gesture recognition. **a** Monitoring a single finger bent to different angles multiple times (∞ means that the finger was continuously bent from 0° to 90° and back to 0°). **b** I–V curves of the GNEC/HAPAAm hydrogel with different bending angles; relationship between bending angle and current at 3.3 V. **c** Hand gesture recognition with the GNEC/HAPAAm hydrogels

The GNEC/HAPAAm hydrogel exhibited excellent sensitivity and responsiveness, and when mounted on the finger, it was used to monitor finger bending motions. As demonstrated in Fig. 6a, the hydrogel accurately detected changes in resistance when the finger was bent from 0 to 90°, and it returned to its initial value when the finger was returned to its original position. Furthermore, the I–V curves were collected for finger bending angles of 0°, 30°, 60°, and 90°, and the relationship between the finger bending angle and current at 3.3 V was verified, which confirmed that the hydrogel accurately distinguished the finger bending angles (Fig. 6b). Finally, five hydrogels were installed on each of the five fingers and used for gesture recognition monitoring (Fig. 6c). The resistance values of the hydrogels installed on the different fingers reflected gesture switching in real-time, demonstrating the great potential of the GNEC/HAPAAm hydrogel for use in flexible and wearable sensors.

## Conclusion

We successfully prepared a GNEC/HAPAAm hydrogel with strong mechanical properties and high sensitivity by integrating a GNEC powder into a hydrophobically associated polyacrylamide hydrogel. The GNEC/HAPAAm hydrogel exhibited the unique advantage of high edge density for the GNEC film, which generated excellent mechanical properties such as a high tensile strain (1685%) and strength (171 kPa), and it served as an elastic contraction device for the underwater robot we designed and fabricated. In addition, the graphene nanocrystals inside the GNEC film formed a conductive network, which caused the hydrogel to respond (150 ms) to external forces. It was used in flexible and wearable sensor applications, such as gesture recognition and human motion monitoring.

## Supplementary information

Supplementary material

## References

[1] Sun, X. et al. Carbon nanotubes reinforced hydrogel as flexible strain sensor with high stretchability and mechanically toughness. *Chem. Eng. J*. **382**, 122832 (2020).

[2] Li MM (2021). Wearable and robust polyimide hydrogel fiber textiles for strain sensors. ACS Appl. Mater. Interfaces.

[3] Ko S, Chhetry A, Kim D, Yoon H, Park JY (2022). Hysteresis-free double-network hydrogel-based strain sensor for wearable smart bioelectronics. ACS Appl. Mater. Interfaces.

[4] Li HJ, Zheng H, Tan YJ, Tor SB, Zhou K (2021). Development of an ultrastretchable double-network hydrogel for flexible strain sensors. ACS Appl. Mater. Interfaces.

[5] Wang Q (2022). Muscle-inspired anisotropic hydrogel strain sensors. ACS Appl. Mater. Interfaces.

[6] Pan SX (2020). Transparent, high-strength, stretchable, sensitive and anti-freezing poly(vinyl alcohol) ionic hydrogel strain sensors for human motion monitoring. J. Mater. Chem. C..

[7] Zeng, J., Dong, L. B., Sha, W. X., Wei, L. & Guo, X. Highly stretchable, compressible and arbitrarily deformable all-hydrogel soft supercapacitors. *Chem. Eng. J*. **383**, 123098 (2020).

[8] Chen K (2021). Effect of directional stretching on properties of PVA-HA-PAA composite hydrogel. J. Bionic. Eng..

[9] Gong JP (2014). Materials both tough and soft. Science.

[10] Dong GH (2021). Starch phosphate carbamate hydrogel based slow-release urea formulation with good water retentivity. Int. J. Biol. Macromol..

[11] Wang RJ (2022). Nanocage ferritin reinforced polyacrylamide hydrogel for wearable flexible strain sensors. ACS Appl. Mater. Interfaces.

[12] Zhu, T. X. et al. A semi-interpenetrating network ionic hydrogel for strain sensing with high sensitivity, large strain range, and stable cycle performance. *Chem. Eng. J*. **385**, 123912 (2020).

[13] Fan, X., Geng, J. H., Wang, Y. L. & Gu, H. B. PVA/gelatin/beta-CD-based rapid self-healing supramolecular dual-network conductive hydrogel as bidirectional strain sensor. *Polymer* **246**, 124769 (2022).

[14] Li MF (2021). Flexible conductive hydrogel fabricated with polyvinyl alcohol, carboxymethyl chitosan, cellulose nanofibrils, and lignin-based carbon applied as strain and pressure sensor. Int. J. Biol. Macromol..

[15] Liang YP, Zhao X, Hu TL, Han Y, Guo BL (2019). Mussel-inspired, antibacterial, conductive, antioxidant, injectable composite hydrogel wound dressing to promote the regeneration of infected skin. J. Colloid Interface Sci..

[16] Liu L (2020). Biomimetic bone tissue engineering hydrogel scaffolds constructed using ordered CNTs and HA induce the proliferation and differentiation of BMSCs. J. Mater. Chem. B.

[17] Lin TY (2022). Temperature-sensitive hydrogels containing carboxylated chitosan-modified carbon nanotubes for controlled drug release. ACS Appl. Nano Mater..

[18] Lu Y (2021). Self-Recovery, fatigue-resistant, and multifunctional sensor assembled by a nanocellulose/carbon nanotube nanocomplex-mediated hydrogel. ACS Appl. Mater. Interfaces.

[19] Zhang WS (2022). Stretchable, self-healing and adhesive sodium alginate-based composite hydrogels as wearable strain sensors for expansion-contraction motion monitoring. Soft Matter.

[20] Zhong Q (2022). Smart DEA-QCGM-CNT hydrogels with temperature- and NIR-responsive behavior achieved by the synergy between CNT and QCGM for wound dressing. Mater. Adv..

[21] Yang C (2017). Reduced graphene oxide-containing smart hydrogels with excellent electro-response and mechanical properties for soft actuators. ACS Appl. Mater. Interfaces.

[22] Wang ZL (2022). 3D printed ultrasensitive graphene hydrogel self-adhesive wearable devices. ACS Appl. Electron. Mater..

[23] Wu, L., Hu, Y. P., Tang, P., Wang, H. & Bin, Y. Z. High stretchable, pH-sensitive and self-adhesive rGO/CMCNa/PAA composite conductive hydrogel with good strain-sensing performance. *Compos. Commun*. **24**, 100669 (2021).

[24] Yu, Y. R., Zhao, X. W. & Ye, L. A novel biocompatible wearable sensors based on poly (vinyl alcohol)/graphene oxide hydrogel with superior self-adhesion, flexibility and sensitivity. *Compos. Struct*. **309**, 116768 (2023).

[25] Du, L. A. et al. Superfast self-healing and photothermal active hydrogel with nondefective graphene as effective additive. *Macromol. Mater. Eng*. **305**, 2000172 (2020).

[26] Chen W (2021). Graphene modified polyaniline-hydrogel based stretchable supercapacitor with high capacitance and excellent stretching stability. Chemsuschem.

[27] Xue P (2023). Highly conductive MXene/PEDOT:PSS-integrated poly(N-isopropylacrylamide) hydrogels for bioinspired somatosensory soft actuators. Adv. Funct. Mater..

[28] Yuan W (2021). MXene-composited highly stretchable, sensitive and durable hydrogel for flexible strain sensors. Chin. Chem. Lett..

[29] Liu YQ (2021). A conductive polyacrylamide hydrogel enabled by dispersion-enhanced MXene@chitosan assembly for highly stretchable and sensitive wearable skin. J. Mater. Chem. B.

[30] Wang SJ (2022). MXene reinforced organohydrogels with ultra-stability, high sensitivity and anti-freezing ability for flexible strain sensors. J. Mater. Chem. C..

[31] Sun, Z. C. et al. Rapid photothermal responsive conductive MXene nanocomposite hydrogels for soft manipulators and sensitive strain sensors. *Macromol. Rapid Comm*. **42**, 2100499 (2021). 10.1002/marc.202100499 34480782

[32] Zhou YH, Fei X, Tian J, Xu LQ, Li Y (2022). A ionic liquid enhanced conductive hydrogel for strain sensing applications. J. Colloid Interface Sci..

[33] Jiao Y (2021). Highly stretchable and self-healing cellulose nanofiber-mediated conductive hydrogel towards strain sensing application. J. Colloid Interface Sci..

[34] Ohm Y (2021). An electrically conductive silver-polyacrylamide-alginate hydrogel composite for soft electronics. Nat. Electron..

[35] Qin ZH (2020). Carbon nanotubes/hydrophobically associated hydrogels as ultrastretchable, highly sensitive, stable strain, and pressure sensors. ACS Appl. Mater. Interfaces.

[36] Wang HF (2021). A highly elastic, room-temperature repairable and recyclable conductive hydrogel for stretchable electronics. J. Colloid Interface Sci..

[37] Wu, G. Z. et al. Fabrication of capacitive pressure sensor with extraordinary sensitivity and wide sensing range using PAM/BIS/GO nanocomposite hydrogel and conductive fabric. *Compos. Part A Appl. Sci. Manuf*. **145**, 106373 (2021).

[38] Wang, Y., Gao, G. H. & Ren, X. Y. Graphene assisted ion-conductive hydrogel with super sensitivity for strain sensor. *Polymer* **215**, 123340 (2021).

[39] Chen SQ (2021). Superstretching MXene composite hydrogel as a bidirectional stress response thixotropic sensor. ACS Appl. Mater. Interfaces.

[40] Zhang X (2023). Direct fabrication of flexible tensile sensors enabled by polariton energy transfer based on graphene nanosheet films. Nanotechnol. Precis. Eng..

[41] Zhang X, Tian LL, Diao DF (2021). High-response heterojunction phototransistor based on vertically grown graphene nanosheets film. Carbon.

[42] Zhang, X., Lin, Z. Z., Peng, D. & Diao, D. F. Bias-modulated high photoelectric response of graphene-nanocrystallite embedded carbon film coated on n-silicon. *Nanomaterials* **9**, 327 (2019). 10.3390/nano9030327 PMC6473602 30823669

[43] Zhang, X. et al. Edge-state-enhanced ultrahigh photoresponsivity of graphene nanosheet-embedded carbon film/silicon heterojunction. *Adv. Mater. Interfaces* **6**, 1802062 (2019).

[44] Lin ZZ, Wang Z, Zhang X, Diao DF (2021). Superhydrophobic, photo-sterilize, and reusable mask based on graphene nanosheet-embedded carbon (GNEC) film. Nano Res..

[45] Zhang X (2022). Direct fabrication of high-performance multi-response e-skin based on a graphene nanosheet film. Soft Sci..

