# Supplementary material for: Bone-inspired (GNEC/HAPAAm) hydrogel with fatigue-resistance for use in underwater robots and highly piezoresistive sensors
Source: Microsyst Nanoeng. 2023 Jul 26;9:99. doi: 10.1038/s41378-023-00571-7 (PMC10368655; doi:10.1038/s41378-023-00571-7)
Supplement: Supplementary file 1 — Supplementary material [file 41378_2023_571_MOESM1_ESM.docx]

**Supplementary information**

**ECR Equipment Introduction**

The ECR nanosurface processing system is composed of a microwave device, an electron cyclotron resonance (ECR) plasma sputtering device, a gas circuit, and a vacuum device. It enables various thin film processing technologies, such as ion and electron irradiation methods. This multifunctional system offers the potential to create and process novel nanosurfaces, thanks to the advantages of both ion and electron irradiation methods, as well as the precise control of irradiation energy and elemental doping content on the substrate surface.

The ECR system provides a high vacuum, clean environment and uses high power microwaves to excite argon gas to generate plasma. The motion of the plasma and electrons is limited by induction of electromagnetic coils, and the electrons are attracted to a 500V target bias. This causes the electrons to bombard the carbon target, sputtering carbon atoms which are gradually deposited on the substrate. By applying a positive bias to the substrate, the electrons are accelerated and collide with the free carbon atoms, causing them to bond with sp2 and form graphene nanocrystals. The ECR system utilizes microwave-induced stable plasma, keeping the internal temperature of the cavity low (100°C-300°C), which facilitates the growth of graphene nanosheet-embedded carbon (GNEC) film on the substrate. By adjusting the parameters of gas flux, microwave power, substrate bias and deposition time during the film growth, GNEC films with different properties can be prepared.

**Pictures of hydrogel sample**


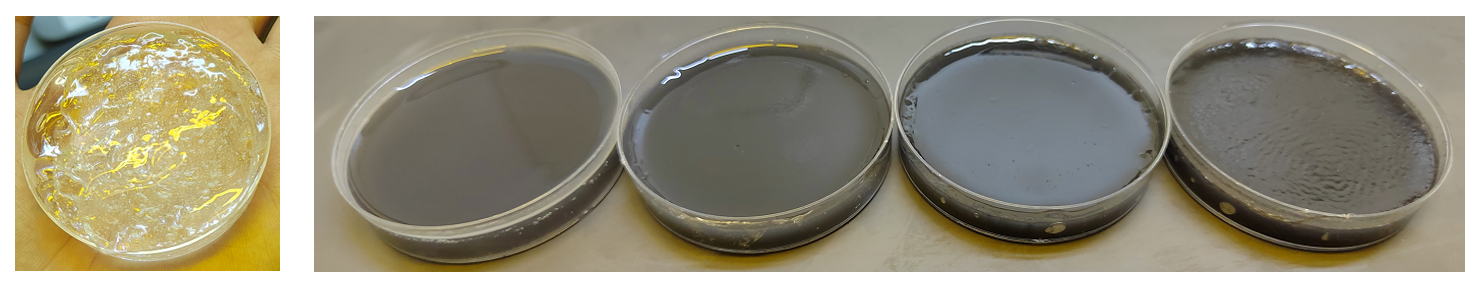


**Fig. S1 HAPAAm hydrogel and GNEC/HAPAAm hydrogel**

As shown in Fig. S1, the hydrogels were just prepared, the GNEC film contents from left to right in the pictures were 0.0wt%, 0.5wt%, 1.0wt%, 1.5wt%, and 2.0wt% respectively. The water content of the hydrogels at this time is high, and the stress-strain curves in Fig. 3a are also measured at this time. The water content can be reduced by drying to obtain better mechanical properties, for example, Fig. 3b, where the water content is 40%.

**Tensile comparison test**


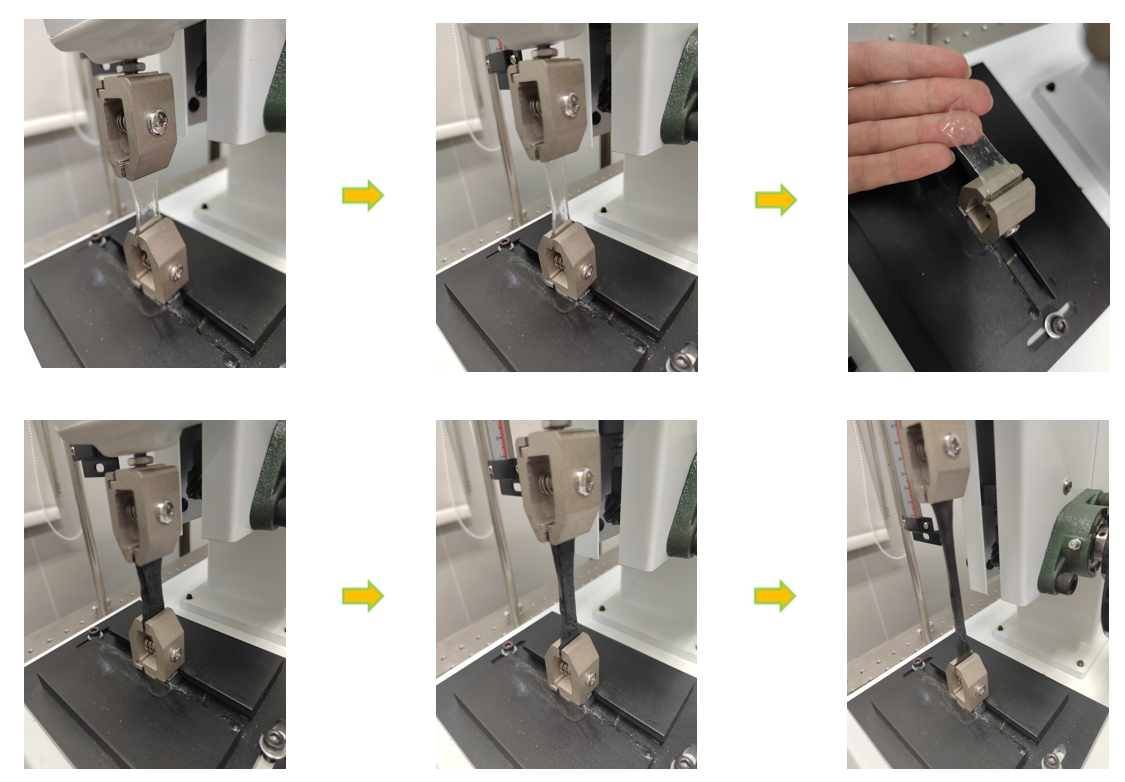


**Fig. S2 Stretching demonstration of HAPAAm hydrogel and GNEC/HAPAAm hydrogel**

Fig. S2 demonstrates the performance of both HAPAAm hydrogel and GNEC/HAPAAm hydrogel. The comparison reveals that the GNEC film can significantly enhances the mechanical properties of the HAPAAm hydrogel.

**Exploded view of underwater robot structure**


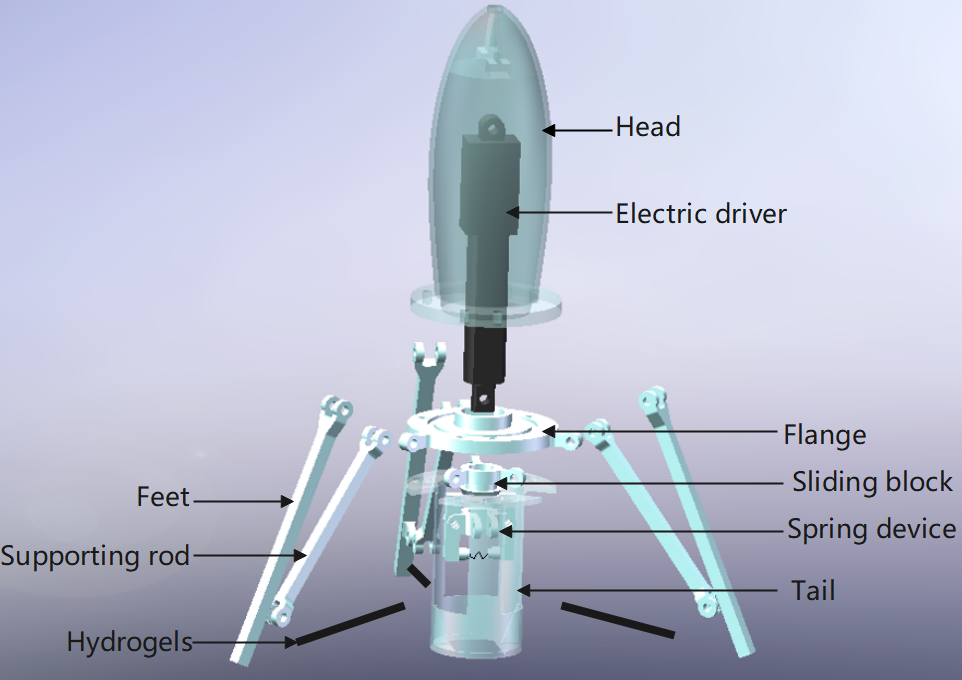


**Fig. S3 The structure of the underwater robot**

As shown in Fig. S3, the feet of the underwater robot rely on an electric actuator to expand and hydrogels to retract, thus completing the swimming motion process through an alternating cycle of expanding and retracting. To ensure a static seal of the flange, a sealing rubber ring is used, while a dynamic seal of the telescopic part of the electric actuator is achieved through the cooperation of a sealing rubber ring and grease.

**Robotics principle supplement**


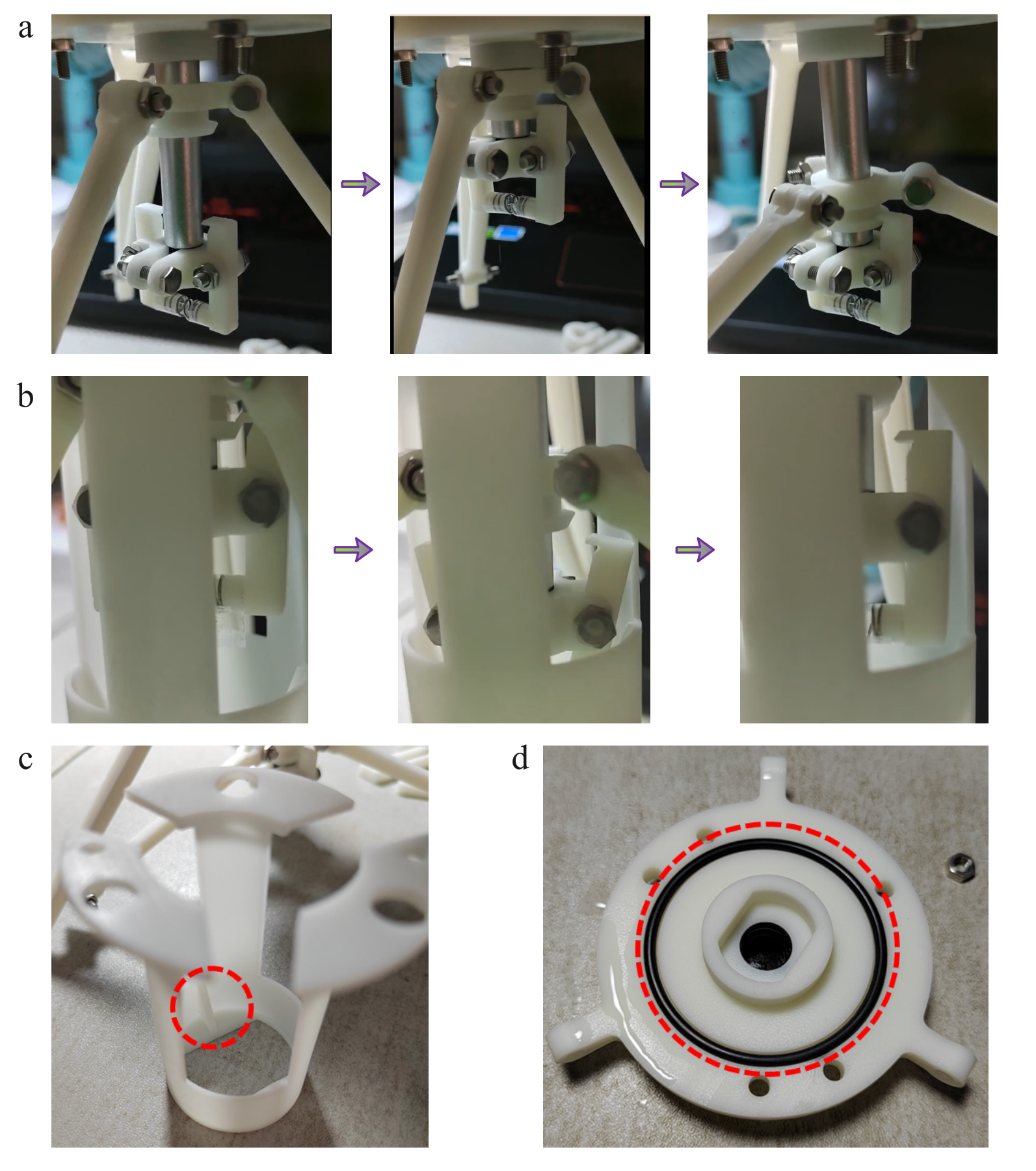


**Fig. S4 Principle of the underwater robot.** **a** Mechanism motion when the robot's foot is open. **b** Mechanism motion when the robot foot is contracted. **c** Ramp structure of the robot's tail sidewall. **d** Installation of the sealing rubber ring.

The principle of the underwater robot motion is supplemented as shown in Fig. S4. The opening of the spring device claws is achieved with the help of the ramp structure shown in Fig. S4c. The GNEC/HAPAAm hydrogel that completes the contraction action is installed with the help of clips and ties.

**Hydrogel Conductivity Test**

**
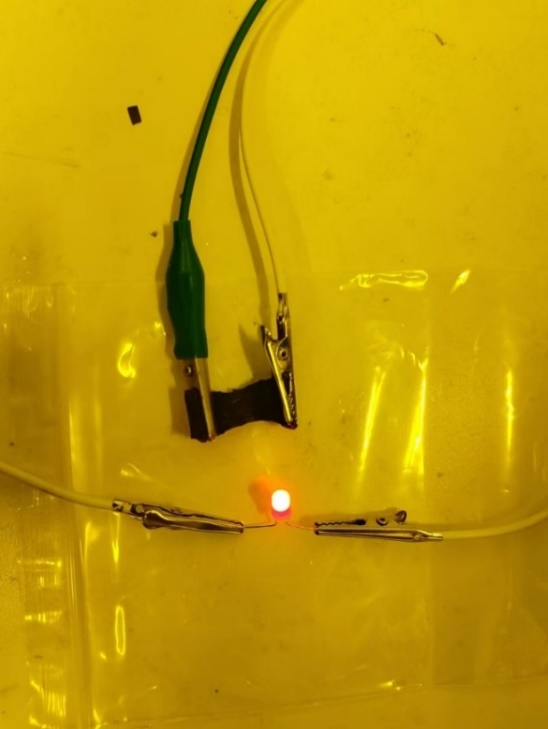
**

**Fig. S5 Conductivity test of hydrogel**

As shown in Fig. S5, the GNEC/HAPAAm hydrogel was successfully connected to the circuit, resulting in the smooth lighting of the LED light.

**Test of hydrogels for underwater sensing applications**


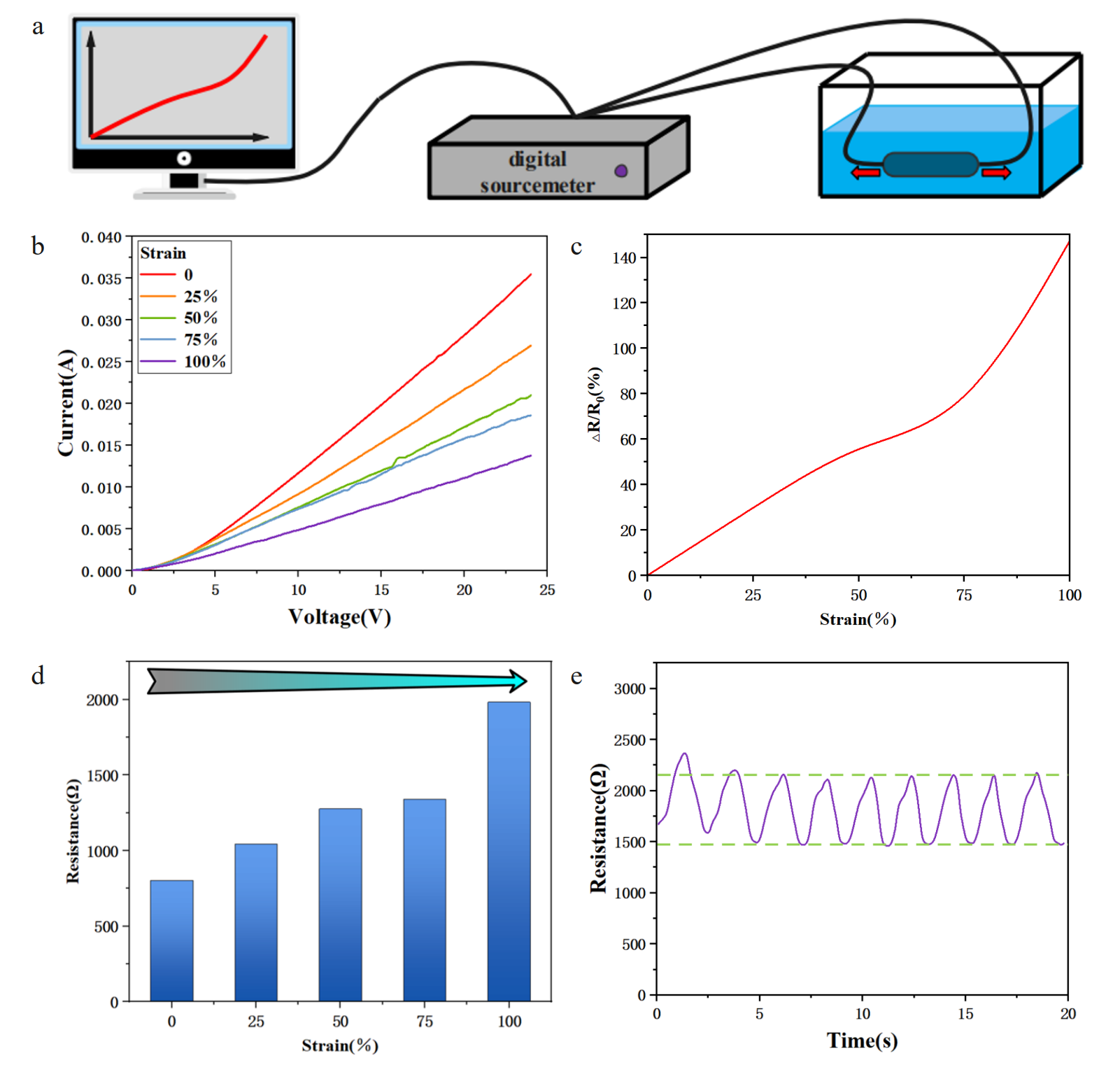


**Fig. S6 Test of hydrogels for underwater sensing applications.** **a** Test system of GNEC/HAPAAm hydrogel for potential application of sensing in underwater environment. **b** I-V curves of the GNEC/HAPAAm hydrogel in underwater environment with different strains. **c** Resistivity variation of the GNEC/HAPAAm hydrogel in the underwater environment with continuous stretching. **d** Resistivity of the GNEC/HAPAAm hydrogel in the underwater environment at different strains. **e** Resistance variation of the GNEC/HAPAAm hydrogel in underwater environment with cyclic stretching.

Since the graphene nanocrystals inside the GNEC film have good electron transport properties and can form a good conductive network, the GNEC film has good electrical conductivity. To verigy its application potential, we tested the hydrogel (GNEC film content=2.0 wt%) by stretching it in the underwater environment (Fig. S6a). As shown in Fig. S6b, we applied voltages to both ends of the hydrogel at different strain states and obtained different I-V curves after the voltage value gradually increased from 0 to 24V, which showed that the electrical properties of the hydrogel varied significantly with strain. As shown in Fig. S6c, we performed continuous stretching of the hydrogel sample and verified that the hydrogel sample had good sensitivity to strain. We also measured the resistance value of the hydrogel at different strain states and found that it increasd from 805 Ω to 1980 Ω, as shown in Fig. S6d. This proved that the hydrogel has potential value for applications in the underwater environment, such as motion monitoring. As shown in Fig. S6e, we also cyclically stretched the hydrogel in the water environment and observed that the resistance value gradually stabilized and fluctuated in the range of 1455 Ω to 2185 Ω. This could be used to monitor the movements of swimmers if overlaid on their joints.

**Test for the effect of temperature on the electrical properties of hydrogels**

In order to verify the effect of temperature on the electrical properties of GNEC/HAPAAm hydrogels, the hydrogels were slowly heated with a heating table. The temperature of the heating table gradually rose from room temperature to 80℃, and the resistance of hydrogels at 40℃, 50℃, 60℃, 70℃ and 80℃ were collected respectively, the result is shown in the Fig. S7. Compared with the resistance of the GNEC/HAPAAm hydrogel itself, the change of the resistance caused by heating was little. It is proved that temperature has little effect on the electrical properties of the GNEC/HAPAAm hydrogels, and the minor fluction in the resistance value should be caused by the water loss of the hydrogels at high temperature. In order to reduce water loss, we can encapsulate the hydrogels with flexible potting adhesive before the experiments.


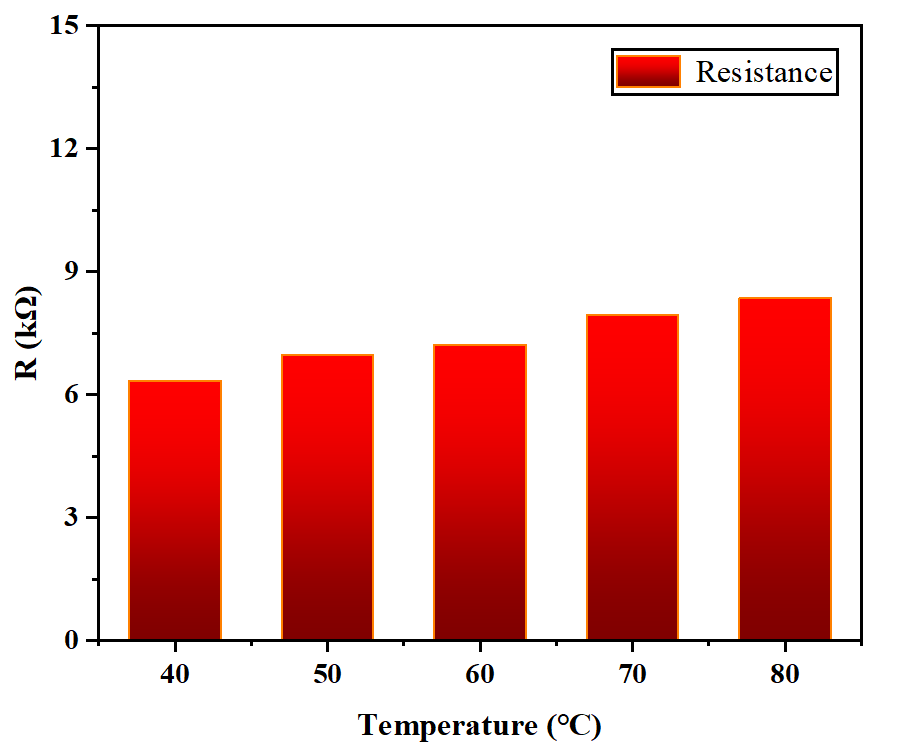


**Fig. S7 The change in the resistance of the hydrogel during heating.**
